# Adhesion Molecule L1 Agonist Mimetics Protect Against the Pesticide Paraquat-Induced Locomotor Deficits and Biochemical Alterations in Zebrafish

**DOI:** 10.3389/fnins.2020.00458

**Published:** 2020-05-28

**Authors:** Thomson Patrick Joseph, Nataraj Jagadeesan, Liu Yang Sai, Stanley Li Lin, Sudhanshu Sahu, Melitta Schachner

**Affiliations:** ^1^Center of Neuroscience, Shantou University Medical College, Shantou, China; ^2^Department of Cell Biology, Shantou University Medical College, Shantou, China; ^3^Guangdong Provincial Key Laboratory for Breast Cancer Diagnosis and Treatment, Shantou University Medical College, Shantou, China; ^4^Keck Center for Collaborative Neuroscience, Department of Cell Biology and Neuroscience, School of Arts and Sciences, Rutgers, The State University of New Jersey, Piscataway, NJ, United States

**Keywords:** pesticide, paraquat, zebrafish, Parkinson’s disease-like pathology, L1 cell adhesion molecule, phenelzine sulfate, tacrine

## Abstract

Besides several endogenous elements, exogenous factors, including exposure to pesticides, have been recognized as putative factors contributing to the onset and development of neurodegenerative diseases, including Parkinson’s disease (PD). Considering the availability, success rate, and limitations associated with the current arsenals to fight PD, there is an unmet need for novel therapeutic interventions. Therefore, based on the previously reported beneficial functions of the L1 cell adhesion molecule, we hypothesized that L1 mimetic compounds may serve to neutralize neurotoxicity triggered by the pesticide paraquat (PQ). In this study, we attempt to use PQ for inducing PD-like pathology and the L1 mimetic compounds phenelzine sulfate (PS) and tacrine (TC) as potential candidates for the amelioration of PD symptoms using zebrafish as a model system. Administration of PQ together with the L1 mimetic compounds PS or TC (250 nM) improved survival of zebrafish larvae, protected them from locomotor deficits, and increased their sensorimotor reflexes. Moreover, application of PQ together with PS (500 nM) or TC (1000 nM) in adult zebrafish counteracted PQ-induced toxicity, maintaining normal locomotor functions and spatial memory in an open field and T-maze task, respectively. Both L1 mimetic compounds prevented reduction in tyrosine hydroxylase and dopamine levels, reduced reactive oxygen species (ROS) generation, protected against impairment of mitochondrial viability, improved the antioxidant enzyme system, and prevented a decrease in ATP levels. Altogether, our findings highlight the beneficial functions of the agonistic L1 mimetics PS and TC by improving several vital cell functions against PQ-triggered neurotoxicity.

## Introduction

Parkinson’s disease (PD) is the second most common and multifactorial progressive neurodegenerative disease that is typically described as being due to dopaminergic (DA) neuron loss in the substantia nigra pars compacta (Dauer and Przedborski, 2003), formation of Lewy bodies (Spillantini et al., 1997), oxidative stress (Jenner, 2003), and mitochondrial and proteasomal dysfunctions (Greenamyre et al., 1999; da Costa, 2003; Ebadi and Sharma, 2003; McNaught and Olanow, 2003). The motor symptoms of PD include bradykinesia, resting tremor, rigidity, and stooped posture that is the outcome of dysregulated basal ganglia circuitries caused by the DA neuron loss (Subramaniam and Chesselet, 2013). Other than motor deficits, sleep disturbances, depression, cognitive deficits, and autonomic and sensory dysfunctions are the non-motor associated symptoms of PD (Perez and Palmiter, 2005; Choi et al., 2008; Chesselet et al., 2012). As far as risk factors are concerned, several lines of evidence highlight the involvement of genetic (Lesage and Brice, 2009; Trinh and Farrer, 2013) and environmental (Tanner et al., 2011) factors, and their joint action (Langston et al., 1984a; Schapira, 2006) in the course of PD, where exposure to pesticides has been well recognized as one of the dangerous environmental factors (Langston et al., 1984b; Schmidt and Ferger, 2001; Ascherio et al., 2006; Costello et al., 2009; Matsui et al., 2009; Tanner et al., 2011; Liew et al., 2014; Chen and Ritz, 2018). A plethora of pesticides has been developed and used to increase agriculture productivity (Muthukumaran et al., 2011), and low-dose exposure of humans to pesticides, in general, and herbicides, in particular, is mostly tolerated by healthy humans. However, long-term exposure and inefficient drug catabolic activity in some individuals leads to certain neurodegenerative diseases (Muthukumaran et al., 2011). Because these chemicals result in oxidative stress and affect mitochondrial metabolism, highly mitochondria-dependent and oxidative stress-sensitive cells, especially neurons, are predominantly affected, resulting in a slow but progressive loss of neurons, and in the onset and development of neurodegenerative diseases (Muthukumaran et al., 2011; Bortolotto et al., 2014; Nellore and Nandita, 2015; Nunes et al., 2017).

In this study, we hypothesized that it may lead to important insights if consequences of the environmental neurotoxin paraquat (PQ) (N,N′-dimethyl-4,4′-bipyridinium chloride), which is a non-selective and highly poisonous herbicide extensively used around the globe (Wesseling et al., 2001; Muthukumaran et al., 2011), could be ameliorated via beneficial compounds. PQ has long been linked to PD in humans (Shults et al., 1998; Couzin, 2007; Tanner et al., 2011; Wu et al., 2012) and acts mainly in DA neurons because of its specificity for the neutral amino acid transporter used by both L-valine and L-dopa (Shimizu et al., 2001; Manning-Boğ et al., 2003). Several animal studies highlight the association of PQ with the loss of DA neurons and thereby increased risk for PD. PQ, when intraperitoneally and repeatedly injected into adult mice, results in the loss of nigral DA neurons in a dose-dependent manner without significantly killing neurons in the striatum (Brooks et al., 1999; McCormack et al., 2002; Thiruchelvam et al., 2003). Furthermore, chronic treatment of rats with PQ results in the progressive loss of DA neurons and a reduction in dopamine levels (Ossowska et al., 2005). Other than in mouse models, PQ, when injected intraperitoneally in adult zebrafish, results in decreased locomotor activity along with impairment of acquisition and consolidation of spatial memory, in parallel with decreased expression of the dopamine transporter gene (Bortolotto et al., 2014) that has been identified as a key player responsible for PQ-mediated neurotoxicity in mice (Rappold et al., 2011). Alterations in the behavior and anti-oxidant defense system of adult zebrafish have been reported upon repeated intraperitoneal injections of PQ (Nunes et al., 2017). In addition, motor deficits at various developmental stages and an increase in oxidative stress have also been reported in zebrafish that were treated with low PQ concentrations (Nellore and Nandita, 2015; Ling et al., 2017). The latest study demonstrated an acceleration of hatch time and impairment of mitochondrial bioenergetics in the early developmental stages after treatment with PQ (Wang et al., 2018). Other than for mice and zebrafish, PQ-mediated DA alterations and motor deficits have been reported in *Caenorhabditis elegans* and *Drosophila* models of PD (Lima et al., 2014; Jahromi et al., 2015).

Regarding therapeutic interventions against PD, existing treatments provide temporary symptomatic relief without restoring mitochondrial function or slowing disease progression (Schapira et al., 2014), and compounds shown to effectively protect DA neurons from 1-methyl-4-phenyl-1,2,3,6 tetrahydropyridine (MPTP)-induced toxicity, *in vivo* and *in vitro*, have so far failed to be neuroprotective in clinical trials (Müller et al., 2003; Investigators, 2007). L-DOPA, which increases DA levels and decreases motor symptoms, continues to be the best treatment currently available for PD by maintaining adequate levels of L-DOPA, and several add-on drugs are often used with ameliorating effects (Schapira et al., 2009; Oertel, 2017). Nevertheless, there is a continued need for treatment strategies that provide neuroprotective and ideally neurorestorative effects in PD. We, therefore, considered to test two L1 agonistic small organic compound mimetics that beneficially act in traumatized nervous system animal models and have been FDA approved for other indications.

Phenelzine sulfate (PS) is a hydrazine derivative, both irreversible and non-selective for monoamine oxidase (MAO) A and B inhibition and has been clinically used for the treatment of several psychiatric disorders (MacKenzie et al., 2010). PS confers neuroprotective effects via elevation of brain gamma-aminobutyric acid (GABA) and brain-derived neurotrophic factor (BDNF) levels, and inhibition of the MAO activity (MacKenzie et al., 2010; Ooi et al., 2015), supporting a potential role for the treatment of neurodegenerative diseases. Consistent with this, a protective effect of PS has been reported for neurons and astrocytes against formaldehyde-induced neurotoxicity by reversion of decreased glutamate uptake via the second messengers Akt and p38 (Song et al., 2010). Furthermore, several lines of evidence highlight the neuroprotective effect of PS in spinal cord (Chen, Z. et al., 2016; Li et al., 2018) and traumatic brain injury (Hill et al., 2019).

Tacrine (TC) (9-amino-1,2,3,4-tetrahydroacridine), a non-selective, reversible cholinesterase inhibitor, affecting acetylcholinesterase and butyrylcholinesterase, was launched as the first drug to alleviate the symptoms of Alzheimer’s disease (Crismon, 1994). Also, it was reported to modulate muscarinic and nicotinic receptor functions and the amyloidogenic pathway (Lahiri et al., 1994; Lahiri et al., 1998; Soukup et al., 2013). TC has also been shown to block MAO activity (Kaul, 1962), neuronal uptake of 5-HT and DA (Clarke et al., 1994), and potassium ion channel activity (Halliwell and Grove, 1989). However, because of its poor oral bioavailability, hepatotoxicity, and gastrointestinal antagonism at the used concentrations, which may have been too high, it has been withdrawn from the market (Watkins et al., 1994; Zeiger et al., 1997; Qizilbash et al., 2000; Reichman, 2003). Therefore, it deemed important to establish a dose–response curve in zebrafish that could form a basis for performing dose–response curves in mammals.

The two L1 mimetic compounds PS and TC were identified by competitive enzyme-linked immunosorbent assay (ELISA) screening of NIH libraries I and II and shown to inhibit binding of the L1 function-triggering 557 monoclonal antibody reacting with the extracellular domain of L1 and to serve as L1 mimetic agonists (Kataria et al., 2016). Therefore, keeping in mind the beneficial roles of L1 and L1 mimetic compounds in other neural diseases (Kataria et al., 2016; Sytnyk et al., 2017; Xu et al., 2017; Li et al., 2018; Sahu et al., 2018), we here attempted to investigate their protective effects against PQ-induced toxicity in zebrafish, which have been well recognized as a useful animal model for other vertebrates because of its evolutionarily conserved brain functions, well-characterized neurotransmitter system, well-described behavioral patterns, and low maintenance costs (Panula et al., 2006; Flinn et al., 2008; Gerlai, 2011). We here demonstrate that the two L1 mimetics overcome PQ-induced deficits in motor and non-motor functions, thus expecting them to be potential additions to the arsenals of compounds treating PD.

## Materials and Methods

### Zebrafish Maintenance

Wild-type adult zebrafish (*Danio rerio*, Strain: AB, 6 months old) were purchased from the Huiyuan Aquatic Animals Company (Shantou, Guangdong, China). Maintenance and breeding were performed according to international guidelines (Westerfield, 2000). Both male and female fish were kept together under the following conditions: light/dark regimen: 14/10 h (ceiling-mounted light tubes, lights “ON” at 8:00 am), temperature: 27 ± 1°C, conductivity: 470–520 μS, pH 6.9–7.2, and feeding twice daily (dried fish food). For breeding, males and females at a ratio of 1:3 were kept in the same tank separated by a vertical sieve. The next day, at dawn upon removal and turning “ON” of the vertical sieve and light, fish begin to mate. After 30–40 min, fish were removed and eggs were collected. The embryos were maintained in embryo medium (E3) (5 mM NaCl, 0.17 mM KCl, 0.33 mM CaCl_2_, and 0.33 mM MgSO_4_, pH 6.8–6.9) (Nusslein-Volhard and Dahm, 2002) at a temperature of 28°C in an incubator until 72 h post-fertilization (hpf). The medium was replaced daily with fresh medium. Experiments in this study were performed in adherence to the protocol reviewed and approved by the Animal Ethics Committee of Shantou University Medical College and by the Director of the Laboratory Animal Center. Attention was paid to keep the number of animals low and to minimize pain.

### Zebrafish Larvae Treatment

**FIGURE 1:**
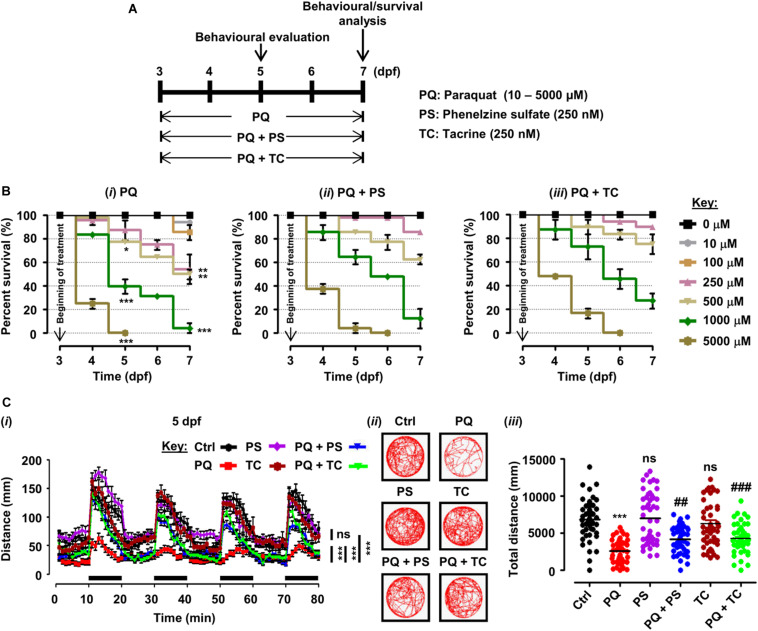
Experimental plan, survival analysis, and behavioral parameters in zebrafish larvae treated with paraquat (PQ) and L1 mimetics. **(A)** Schematic representation of the experimental plan for zebrafish larvae treatment with PQ and L1 mimetics. At 3 dpf, morphologically normal larvae were treated with the indicated concentrations of PQ and at the same time exposed to phenelzine sulfate (PS) and tacrine (TC) until 7 dpf. At 5 and 7 dpf, a behavioral evaluation was performed while survival under different treatment conditions was investigated until 7 dpf. **(B)** Survival analysis of zebrafish larvae after treatment with **(*i*)** PQ (10–5000 μM), **(*ii*)** PQ (10–5000 μM) + PS (250 nM), and **(*iii*)** PQ (10–5000 μM) + TC (250 nM) until 7 dpf. *p < 0.05, **p < 0.01, and ***p < 0.001 PQ *vs.* control group. **(C)** Spontaneous movement analysis of zebrafish larvae under treatment with PQ (500 μM) and L1 mimetics (250 nM) from 3 dpf until measurements (5 dpf). **(*i*)** Vertical axis shows the average distance traveled by larvae in a 1-min bin under alternate 10-min light and dark (black bars on the *x*-axis) conditions for 80 min, ***p < 0.001 PQ *vs.* control group and PQ *vs.* PQ + PS or PQ + TC groups; ns, not significant PS or TC *vs.* control group; **(*ii*)** representative traces of individual larva in the swimming test; **(*iii*)** *y*-axis shows the total distance traveled by larvae in a period of 80 min, ***p < 0.001 PQ *vs.* control group, ^##^*p* < 0.01, and ^###^*p* < 0.001 PQ *vs.* PQ + PS and PQ + TC groups; ns, not significant PS or TC *vs.* control group. Data are presented as mean ± SEM of two independent experiments (*n* = 24 larvae/group/experiment) and analyzed by one-way analysis of ANOVA using Tukey’s *post hoc* test.

For the initial, less time-consuming and thus the efficient investigation of the role of L1 mimetics in PQ-induced toxicity, the early-life stage of zebrafish was used and its impact on survival and behavior was determined. The experimental plan for zebrafish larvae is presented in Figure 1A. The groups under different treatment conditions are abbreviated as follows: Control, Ctrl; paraquat, PQ; phenelzine sulfate, PS; tacrine, TC; paraquat + phenelzine sulfate, PQ + PS; paraquat + tacrine, PQ + TC. For treatment, stock solutions of PQ dichloride (C_12_H_14_Cl_2_N_2_, purity 99.7%, Cat no. P814066, *Macklin* Biochemical Co., Ltd., Shanghai, China), PS (C_8_H_14_N_2_O_4_S, 99.98%, Cat no. HY-B1018A, MedChemExpress, NJ, United States), and TC (C_13_H_17_ClN_2_O, 98.70%, Cat no. HY-B2244, MedChemExpress, NJ, United States) were prepared in distilled water, and the exposure solutions were prepared by diluting stock solutions into E3 media to achieve the desired concentrations.

For survival analysis, hatched larvae at 3 dpf without apparent abnormalities were exposed to PQ (10–5000 μM) and L1 mimetics (PS and TC) in a six-well plate (Jet Biofil, Guangzhou, China) (24 larvae/3 ml/well) until 7 dpf. The exposure tests were conducted following the OECD guidelines (OECD, 1992), the concentration range of PQ was selected based on previous studies (Imamura et al., 2011; Wang et al., 2018; Pinho et al., 2019), and for L1 mimetics (250 nM), concentration was selected for both mimetics based on their beneficial role in previous studies (Li et al., 2018; Sahu et al., 2018). In order to determine the effect of different treatments on the survival of zebrafish larvae, each concentration (10–5000 μM) of PQ alone and PQ in the presence of L1 mimetics (PS or TC) at 5 and 7 dpf were individually compared and statistically analyzed by one-way analysis of variance (ANOVA) using Tukey’s *post hoc* test. For evaluation of behavioral experiments, such as spontaneous swimming and sensorimotor reflexes, zebrafish larvae were exposed to different treatment conditions from 3 to 5 or 7 dpf in six-well plates (30–40 larvae/5 ml/well). In all experiments, larvae were observed daily under a stereomicroscope (Model no. MSV269, Leica, Taipei, Taiwan) to assess vitality. Dead larvae (absence of heartbeat) were removed, and treatment solutions were replaced daily with fresh solutions.

### Evaluation of Behavior

Larvae were observed after treatment under a stereomicroscope (Model no. MSV269, Leica, Taipei, Taiwan) to detect deformities (spinal aberrations and loss of equilibrium), and normomorphic larvae were transferred to a 24-well plate for measurements of spontaneous swimming at 5 and 7 dpf. The plate was placed in the ZebraBox equipment (ViewPoint Life Science, Lyon, France), and the larvae were acclimatized to the chamber for 30 min before the test. Locomotor activities of 24 larvae from control and different treatment groups were evaluated in terms of total distance swum for 80 min, with measurements consisting of alternating 10-min periods of light and dark, using a video camera and an infrared light plus filter installed in the observation chamber. The ZebraBox equipment-generated spreadsheets (Excel, Microsoft) were used to derive movement parameter: total distance traveled (mm).

After measurements of spontaneous swimming, each larva was gently touched on head and tail with a micropipette tip to test sensorimotor reflexes. Immediate swimming was considered a positive response, whereas no response was considered as a negative response, with results being presented as percent head and tail reflexes (Pinho et al., 2016, 2019).

### Treatment of Adult Zebrafish With PQ and L1 Mimetic Agonists

**FIGURE 2:**
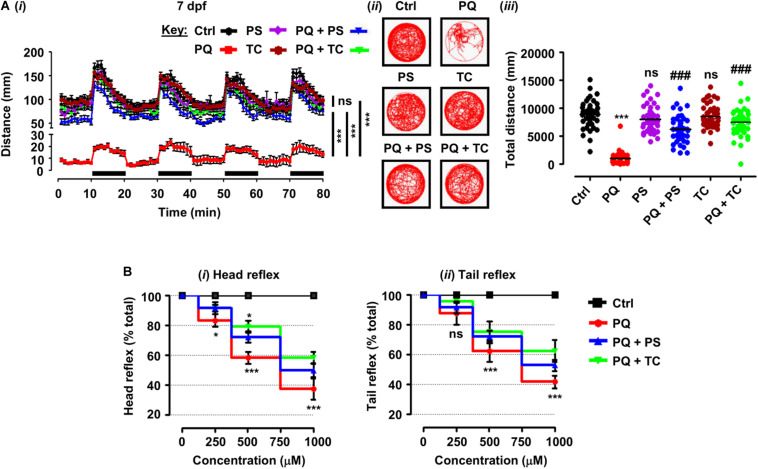
Behavioral parameters and sensorimotor reflexes in zebrafish larvae under different treatment conditions. **(A)** Spontaneous movement analysis of zebrafish larvae under treatment with paraquat (500 μM) and L1 mimetics (250 nM) from 3 dpf until measurements (7 dpf). **(*i*)** Vertical axis shows the distance traveled by larvae in a 1-min bin under alternate 10-min light and dark (black bars on the *x*-axis) conditions for 80 min, ***p < 0.001 PQ *vs.* control group and PQ *vs.* PQ + PS or PQ + TC groups; ns, not significant PS or TC *vs.* control group; **(*ii*)** representative traces of individual larva in the swimming test; **(*iii*)** *y*-axis shows the total distance traveled by larvae in a period of 80 min, ***p < 0.001 PQ *vs.* control group, ^###^*p* < 0.001 PQ *vs.* PQ + PS and PQ + TC group; ns, not significant PS or TC *vs.* control group. **(B)** Sensorimotor reflexes at 5 dpf: **(*i*)** head reflexes and **(*ii*)** tail reflexes after exposure to paraquat (250, 500, and 1000 μM) and L1 mimetics (250 nM), *p < 0.05, ***p < 0.001; ns, not significant. Data are presented as mean ± SEM of two independent experiments (*n* = 24 larvae/group/experiment) and analyzed by one-way ANOVA using Tukey’s *post hoc* test.

For the detailed and sex-specific investigation regarding PQ and effect of L1 mimetics, male adult zebrafish were used, as the occurrence of PD is more frequent in males than females (Cerri et al., 2019). The detailed experimental paradigm for adult zebrafish is presented in Figure 2A. Stock solutions of PS and TC were dissolved in distilled water and then diluted in aquarium water to the desired concentrations. For the L1 mimetic compounds, previously reported beneficial concentrations in adult zebrafish were considered (Li et al., 2018; Sahu et al., 2018). After the acclimatization period, adult male zebrafish (0.4–0.6 g) were anesthetized by immersion in a solution of 0.033% MS222 (ethyl 3-aminobenzoate methane sulfonate) (Cat no. 886-86-2, Sigma–Aldrich, St. Louis, MO, United States), injected with PQ (20 mg/kg/≤5 μl) intraperitoneally for a total of six injections (one injection every 3 days) as performed previously (Bortolotto et al., 2014), while being exposed at the same time to 500 nM of PS or 1000 nM of TC in the tank water for behavioral experiments. The control group received an equal volume (≤5 μl) of 0.9% saline vehicle solution. All exposure solutions were changed once daily.

### Analysis of Behavior

#### Locomotion

Locomotion was analyzed 24 h after the final treatment for each group (control, PQ, L1 mimetic, and PQ + L1 mimetic) in the open field (Champagne et al., 2010). Fish were placed individually into an experimental tank (42 cm × 30 cm × 30 cm) filled with aquarium water (5 cm deep). After habituation for 2 min, total distance traveled was evaluated for 6 min using Ethovision (Noldus, Wageningen, Netherlands).

The total distance swum was defined as the distance covered by the fish in a time bin of 6 min and is represented in centimeters. Additionally, we also analyzed the number and durations of freezing bouts. A period of immobility for at least 2 s was operationally defined as freezing, as characterized by frequent operculum movements (Müller et al., 2018).

#### T-Maze Task

The spatial memory was analyzed after treatments in a T-maze following the method for the Y-maze (Bortolotto et al., 2014). In this task, zebrafish were individually trained and tested in a T-maze aquarium with three arms (24 cm × 6 cm × 15 cm) that were designated as the start arm, open arm (that is always open), and the novel arm (that is closed in the training session but opened in the test session). In the training session (5 min), the novel arm was kept closed, and zebrafish were placed into the start arm and allowed to explore the maze for 5 min. After 1 h of training, the test session (5 min) was started during which the novel arm was opened, and zebrafish were placed into the start arm and allowed to explore the maze again. The time spent in each arm was determined. Both the training and test sessions were evaluated by Ethovision.

### Biochemical Analysis

#### Brain Tissue Preparation

After treatment under different conditions, adult zebrafish were anesthetized by immersion in 0.033% MS222 and euthanized by punching the spinal cord behind the opercula. Brains were removed and immediately placed on ice, washed with 0.9% saline, transferred to microtubes, and stored at −80°C until use. All biochemical analysis was performed in triplicate with pooled samples of five brains/group.

#### Western Blot Analysis for Tyrosine Hydroxylase (TH)

Brains (*n* = 5/group) from each treatment group were used for Western blotting. Briefly, brains were pooled and lyzed in 300 μl of RIPA buffer (Cat no. R0010, Solarbio Science and Technology Co., Ltd., Beijing, China) supplemented with 1 mM of phenylmethylsulfonyl fluoride (PMSF) (Cat. no. B111-01, GenStar, Shanghai, China) and 1% phosphatase inhibitors (Cat. no. P1260, Solarbio Science and Technology Co., Ltd., Beijing, China). Lysis was facilitated by using a tissue homogenizer (Cat no. G55500-0000, Dogger Instruments Co., Taipei, Taiwan) on ice. Then, 40 μg of extracted protein was separated by 10% SDS/PAGE and transferred to polyvinylidene fluoride (PVDF) membranes (Cat no. IPVH00010, Merck Millipore Ltd., Tullagreen, Carrigtwohill, Country Cork, Ireland). In order to prevent non-specific antibody binding, membranes were treated with blocking buffer [5% bovine serum albumin (Cat no. A8020, Solarbio Science and Technology Co., Ltd., Beijing, China) in TBS-T (20 mM Tris–HCl (pH 7.5), 150 mM NaCl, 0.1% Tween 20)] at room temperature for 1 h. Blots were incubated at 4°C overnight with the following antibodies diluted in blocking buffer: TH (1:500, Cat no. 25859-1-AP, Proteintech, Wuhan, China) and β-actin (1:1000, Cat. no. BM0626, Boster Biological Technology, Wuhan, China). After three washes with TBS-T, membranes were incubated with the secondary antibodies: goat anti-rabbit IgG (HRP-conjugated, 1:1000 Cat. no. BA1055, Boster Biological Technology, Wuhan, China) or goat anti-mouse IgG (HRP-conjugated, 1:1000 Cat. no. BA1051, Boster Biological Technology, Wuhan, China) at room temperature for 1 h. After three washes with TBS-T, blots were developed with enhanced chemiluminescence (ECL) detection kit (Cat no. 1705060, Bio-Rad Laboratories, Inc., CA, United States) and images were captured with a FluorChem^®^ Q imaging system (Alpha Innotech, San Leandro, CA, United States).

#### Enzyme-Linked Immunosorbent Assay (ELISA) for the Determination of DA Levels

Levels of DA in brain samples (*n* = 5/group) were determined by using a commercially available ELISA kit (Cat no. ZG-E1753, Zgenebio Company, Taipei, Taiwan) following the manufacturer’s instructions. Briefly, brains were homogenized using phosphate-buffered saline (PBS) pH 7.2, and total protein concentration was determined. Then, 10 μg of each sample was used for the assay. After terminating the reaction with stop solution, color intensity was measured within 15 min at 450 nm using an Infinite M200 PRO multimode reader (Tecan, Austria), and the concentration of DA in the samples was then determined by comparing the OD of the samples to the standard curve. Data are expressed as pg/μg of total protein.

#### Biomarker of Oxidative Damage

Measurement of reactive oxygen species (ROS) levels in the brain samples (*n* = 5/group) was performed according to the manufacturer’s instructions using the ROS determination kit (Cat no. E004, Nanjing Jiancheng Bioengineering Institute, Nanjing, China). After treatment, single-cell suspensions of the tissue were prepared by using a glass mortar and pestle followed by pipetting up and down using Pasteur pipettes until the tissue appeared well dispersed. A nylon mesh was used to remove tissue junks. The homogenate was centrifuged at 1000 *g* for 10 min, and the cell pellet was then exposed to 10 μM of 2′,7′-dichlorofluorescein diacetate (DCFH-DA) solution at 37°C for 30 min. After two PBS washes, cells were resuspended in 0.5 ml PBS, and the fluorescence intensity (Ex 500 ± 15 nm/Em 525 ± 20 nm) was analyzed by an Infinite M200 PRO multimode reader (Tecan, Austria). Fluorescence intensity is proportional to the intracellular ROS level and presented as a percent of control.

#### Antioxidant Enzymes

After treatment, total superoxide dismutase (T-SOD) activity in brains (*n* = 5/group) was determined by the xanthine oxidase assay, based on its ability to reduce superoxide anion radicals, using the T-SOD kit (Cat no. A001-1, Nanjing Jiancheng Bioengineering Institute, Nanjing, China). The color intensity was measured at 550 nm using the M200 PRO multimode reader. Results are expressed as enzyme units per milligram of protein (U/mg).

#### Mitochondrial Viability

For the evaluation of mitochondrial viability, brains were stained with 2,3,5-triphenyltetrazolium chloride (TTC) (Cat no. T8170, Solarbio Science and Technology Co., Ltd.) following an established protocol (Preston and Webster, 2000; Braga et al., 2013; Nunes et al., 2017). Brains (*n* = 5/group) were exposed to 2% TTC at 37°C for 40 min under protection from light. The reaction was stopped with 10% formalin (Cat no. 50-00-0, Xilong Scientific, Shantou, China). The 2% TTC and 10% formalin solutions were prepared in phosphate buffer (pH 7.4). Brains were then dried at 40°C, weighed, and immersed in 200 μl of dimethylsulfoxide (DMSO) (Cat no. 67-68-5, Generay Biotech Co., Ltd., Shanghai, China) under constant agitation for 4 h to elute the formazan produced by the TTC reaction. Absorbance of the supernatant was determined at 490 nm using the Infinite M200 PRO multimode reader. Data are presented as absorbance per tissue dry weight in (g) and normalized as a percentage of control.

#### Measurement of ATP

ATP levels in brain samples (*n* = 5/group) were determined by the luciferase-based enhanced ATP assay kit (Cat no. S0027, Beyotime Biotechnology, Shanghai, China) following the manufacturer’s instructions. Luminescence was detected within 30 min after brain dissection using the Infinite M200 PRO multimode reader. The concentration of ATP was calculated according to an ATP standard curve in nmol/mg, and results are expressed as percent of control.

#### Protein Quantification

The bicinchoninic acid (BCA) protein assay kit (Cat no. E162-01, GenStar, Beijing, China) was used according to the manufacturer’s instructions. Absorbance was measured at 562 nm.

### Statistical Analysis

The number of animals (represented by *n*) used in each experiment, statistics test, and *p*-values are presented in the figures or figure legends. For comparison of multiple groups, one-way ANOVA was used, followed by Tukey’s multiple comparison test. Data fittings and statistical analyses were performed with Prism V5.0 software (GraphPad Software, Inc., United States). Data are presented as mean ± standard error of the mean (SEM), and the comparison level of significance was defined as *p* < 0.05.

## Results

### L1 Mimetics Protect From PQ-Triggered Locomotor Deficits in Zebrafish Larvae

Zebrafish larvae, when treated with different concentrations of PQ (10–5000 μM) from 3 to 5 and 7 dpf, showed a dose- and time-dependent reduction in survival (Figures 1A,B*i*). At 5 dpf, numbers of surviving larvae were reduced to 77.08% (*p* < 0.05) by 500 μM, and 39.58% (*p* < 0.001) by 1000 μM, and further reduced to 50% (*p* < 0.01), and 4.16%, (*p* < 0.001), respectively, at 7 dpf in comparison with untreated controls. At 5 dpf, 5000 μM PQ resulted in 100% mortality, and at 7 dpf, the LD_50_ of PQ was determined to be approximately 500 μM.

In order to evaluate the beneficial role of L1 mimetics (PS and TC) in PQ-treated zebrafish, larvae were exposed to different concentrations of PQ (10–5000 μM) together with 250 nM PS or TC in E3 medium (Figure 1A). At 5 and 7 dpf, PS treatment did not significantly increase survival of larvae treated with 500 μM PQ (from 77.08 to 85.42%), 1000 μM PQ (from 39.58 to 64.58%) and 500 μM PQ (from 50 to 62.50%), 1000 μM PQ (from 4.16 to 12.50%), respectively (Figure 1B*ii*). TC treatment also did not significantly increase survival at 5 and 7 dpf at 500 μM (from 77.08 to 89.58%), 1000 μM (from 39.58 to 72.92%) and 500 μM (from 50 to 75%), 1000 μM (from 4.16 to 27.08%) (Figure 1B*iii*).

To elucidate the beneficial role of L1 mimetics in the *in vivo* Parkinsonian-like phenotype induced by PQ, zebrafish larvae were treated with the LD_50_ (500 μM) of PQ together with 250 nM of PS or TC and then assessed for sensorimotor reflexes at 5 dpf and spontaneous swimming at 5 and 7 dpf. After the 30-min habituation period, the activity of each larva was recorded for 80 min over four alternate light/dark cycles (each consisting of 10 min). PQ reduced the distance swum (*p* < 0.001) at 5 dpf (Figures 1C*i–iii*) and 7 dpf (Figures 2A*i–iii*) when compared to the untreated control. Co-treatment with either PS or TC (250 nM) prevented the toxic effects of PQ regarding distance swum both at 5 dpf (Figures 1C*i–iii*) and 7 dpf (Figures 2A*i–iii*). The co-treated larvae both at 5 and 7 dpf moved as well as the untreated control, and significantly more than the PQ-treated group (PS: *p* < 0.001 *vs.* PQ, TC: *p* < 0.001 *vs.* PQ), demonstrating the beneficial effect of the L1 mimetics. Besides, no significant effects were observed upon L1 mimetic (PS or TC) treatment alone in comparison with the untreated control group. In addition, larvae after a 48-h (3–5 dpf) treatment with PQ (250, 500, and 1000 μM) showed a concentration-dependent reduction in the escape response evoked either by head- or tail-touch stimulation (Figures 2B*i,ii*), indicating impaired sensorimotor reflexes. In agreement with a protective effect of L1 mimetics in a spontaneous swimming test, both compounds also showed improved sensorimotor responses in both the head- and tail-touch evoked test when larvae were exposed to PQ (250, 500, and 1000 μM) together with the L1 mimetic. TC significantly (*p* < 0.05) improved the sensorimotor reflexes for head-touch stimulation at a PQ concentration of 500 μM.

### L1 Mimetics Ameliorate PQ-Triggered Locomotor and Exploratory Deficits in Adult Zebrafish

**FIGURE 3:**
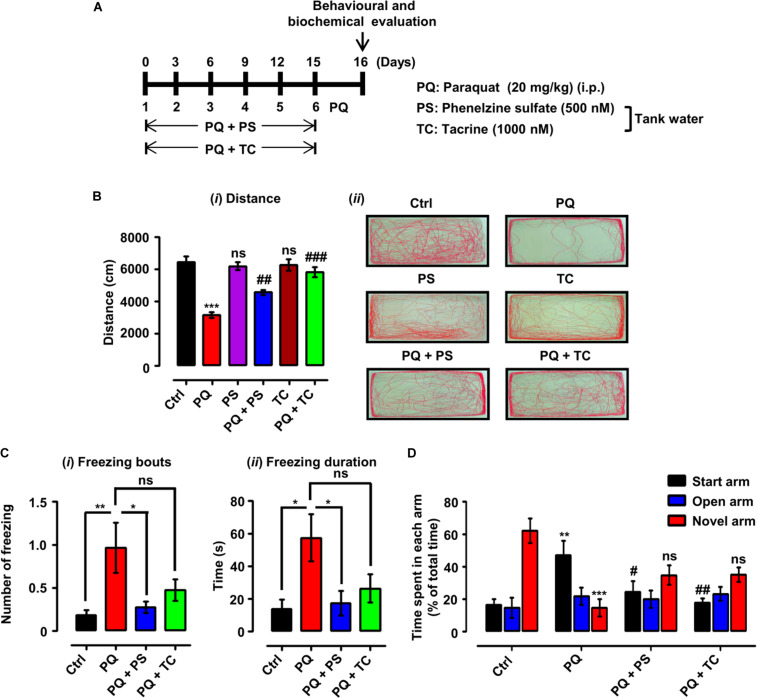
Experimental scheme and behavioral parameters in adult zebrafish treated with paraquat (PQ) and L1 mimetics. **(A)** For the protective investigations of L1 mimetics, adult zebrafish after an acclimation period of 7 days were divided into four groups. The control group (Ctrl) was given an equal volume of saline intraperitoneally (i.p.), the PQ group was treated with 20 mg/kg of PQ via intraperitoneal injection (i.p.), and the PQ + L1 mimetics group (PQ + PS/PQ + TC) were given PQ [i.p. (20 mg/kg)] and exposed to L1 agonists (PS 500 nM, TC 1000 nM) at the same time in the tank water. The short vertical lines represent the day of PQ injection, which was administered every 3 days (six injections in total) over 15 days, and 24 h after last injection behavioral and biochemical evaluation was done. **(B)** Locomotor profile of adult zebrafish under different treatment conditions. 24 h after the last treatment, total distance traveled was evaluated. **(*i*)** Total distance traveled and **(*ii*)** representative traces of individual zebrafish in a period of 6 min. **(C)** Open field 24 h after different treatments, showing the results of **(*i*)** number of freezing bouts and **(*ii*)** freezing duration, *p < 0.05 and **p < 0.01; ns, not significant. **(D)** T-maze response to novelty in control (saline-treated)-, PQ-, and L1 mimetic-treated groups, **p < 0.01, ***p < 0.001 PQ vs control group, ^##^*p* < 0.01 PQ vs PQ + PS, ^###^*p* < 0.001 PQ vs PQ + TC group and ns, not significant PS or TC vs control group. Data are expressed as mean ± SEM of three independent experiments (*n* = 15–25 animals/group/experiment) and were analyzed by one-way analysis of ANOVA using Tukey’s *post hoc* test.

Twenty-four hours after the last treatment with indicated concentrations of PQ and L1 mimetic (Figure 3A), the locomotor and exploratory activities were recorded. In the open field, treatment of adult zebrafish with PQ at a concentration of 20 mg/kg intraperitoneally resulted in a reduction in the total distance swum (*p* < 0.001) in comparison with the control saline-injected group (Figure 3B*i*). In comparison to the PQ-treated zebrafish, L1 mimetic-treated groups were protected from the effects of PQ [(PQ + PS, *p* < 0.01) (PQ + TC, *p* < 0.001)], while no significant differences were observed between PS or TC alone and the saline-injected control group. In addition, PQ-treated zebrafish avoided exploring the center of the tank and preferred to swim near the walls in comparison with the saline-injected control group, highlighting the anxiety-like behavior induced by PQ treatment. L1 mimetics prevented the induction of anxiety-like behavior in PQ + PS- and PQ + TC-treated groups (Figure 3B*ii*). Similarly, one-way ANOVA showed an increase in the number of freezing bouts (*p* < 0.01) and freezing durations (*p* < 0.05) in the PQ-treated group when compared to the control group. These effects were reduced by PS, reflected in the PQ + PS-treated group (*p* < 0.05), but not significantly reduced in the PQ + TC-treated group in comparison to the PQ-treated group (Figures 3C*i,ii*).

The effect of PQ and L1 agonist on spatial memory was evaluated by subjecting the zebrafish to the T-maze task, in which zebrafish are given training, and after 1 h of training, time spent in each arm (start, open, and novel arm) and the response to novelty (time spent in the novel arm) were determined. The PQ-treated group spent less time in the novel arm (*p* < 0.05) and more time in the start arm (*p* < 0.01) in comparison to the control group. However, PQ-treated zebrafish in combination with PS or TC did not significantly spend more time in the novel arm and significantly less time in the start arm [(PQ + PS, *p* < 0.05) (PQ + TC, *p* < 0.01)] in comparison to the PQ-only group (Figure 3D).

### PQ-Induced Biochemical Alterations Are Inhibited by PS and TC

**FIGURE 4:**
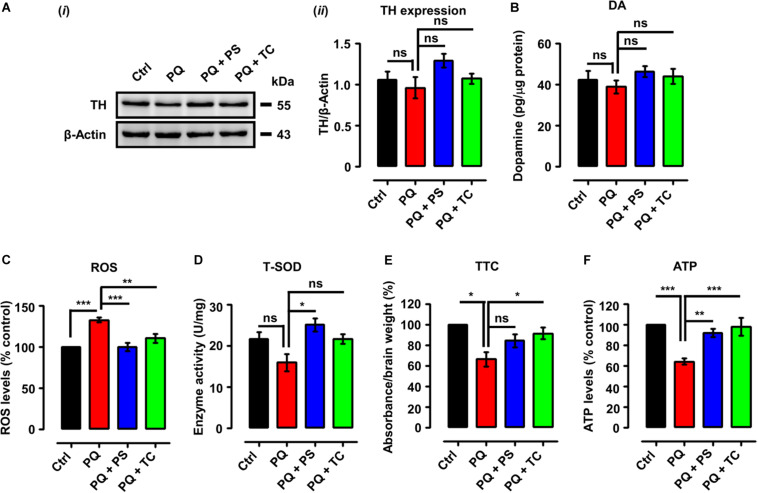
Biochemical parameters in adult zebrafish brain after treatment with paraquat and L1 mimetics. **(A) (*i*)** Western blot analysis of tyrosine hydroxylase (TH) protein and **(*ii*)** quantification of Western blots. **(B)** Dopamine (DA) levels. **(C)** Reactive oxygen species (ROS) levels. **(D)** Total superoxide dismutase (T-SOD) activity. **(E)** Mitochondrial viability assay using 2,3,5-triphenyltetrazolium chloride (TTC). **(F)** ATP levels. Data are presented as mean ± SEM of three independent experiments (*n* = 5 brains/group/experiment) and analyzed by one-way analysis of variance (ANOVA) using Tukey’s *post hoc* test, *p < 0.05, **p < 0.01, and ***p < 0.001; ns, not significant.

We next tested if the recovery of PQ-induced deficits in locomotor and exploratory behavior by the L1 mimetic is associated with neurochemical changes. We determined TH expression and DA levels in adult brains in the different treatment groups. One-way analysis of ANOVA revealed no significant differences among the groups for both TH expression (Figures 4A*i,ii*) and DA levels (Figure 4B). However, there was a tendency to decrease TH and DA expression levels upon treatment with PQ and L1 mimetics.

Given the pro-oxidant properties of PQ, we determined ROS levels following treatment with PQ with or without PS or TC. One-way ANOVA indicated an increase of ROS levels in the PQ-treated group (*p* < 0.001), in comparison to the control group, which was prevented by PS (*p* < 0.001) and TC (*p* < 0.01) (Figure 4C).

We also investigated an anti-oxidant parameter, which revealed a reduction of T-SOD levels in the PQ-treated group (Figure 4D) in comparison to the control. PS increased T-SOD levels (*p* < 0.05), while TC treatment prevented the reduction in T-SOD levels when compared to the PQ group.

The effect of PQ and L1 mimetics on mitochondrial function was determined by TTC staining. Quantification of formazan depicted a reduction in mitochondrial viability in the PQ-treated group (*p* < 0.05), when compared to the control group, which was prevented to some extent by PS and significantly by TC (*p* < 0.05) (Figure 4E). To further investigate the effect of PQ on mitochondrial function, we determined ATP levels. Upon PQ treatment, a reduction in ATP levels was observed in comparison to the saline-treated control group (*p* < 0.001). The groups that were co-treated with PS (*p* < 0.01) or TC (*p* < 0.001) showed increased ATP levels in comparison to the PQ-treated group (Figure 4F).

## Discussion

The neural cell adhesion molecule L1CAM has been reported to have functional roles in the developing and adult nervous system, and a knock-out mouse mutation of L1 has been associated with the L1 syndrome (Kamiguchi et al., 1998; Hortsch, 2000; Loers and Schachner, 2007; Maness and Schachner, 2007; Schmid and Maness, 2008; Schafer and Altevogt, 2010; Hortsch et al., 2014; Sytnyk et al., 2017). Several lines of evidence highlight a link of L1 to neurodegenerative diseases (Fransen et al., 1995, 1996; Poltorak et al., 1995; Kurumaji et al., 2001; Strekalova et al., 2006; Maness and Schachner, 2007; Schmid and Maness, 2008; Wakabayashi et al., 2008; Schafer and Altevogt, 2010). L1.1 and L1.2, orthologs of mammalian cell adhesion molecule, contribute to the regeneration of the spinal cord in adult zebrafish (Becker et al., 2004; Chen, T. et al., 2016). In other studies, the beneficial role of L1 mimetics in spinal cord injury through L1 signaling in mice and zebrafish has been described (Li et al., 2018; Sahu et al., 2018). We now extended these studies to investigate in zebrafish the role of two already reported agonistic L1 mimetic compounds (Kataria et al., 2016), PS and TC, as potential protective agents against PQ-induced behavioral deficits and biochemical alterations. Several animal studies highlight the close resemblance of PQ-induced behavioral and neurochemical impairments with those observed in PD (Brooks et al., 1999; McCormack et al., 2002; Thiruchelvam et al., 2003; Ossowska et al., 2005; Bortolotto et al., 2014; Lima et al., 2014; Jahromi et al., 2015; Nellore and Nandita, 2015; Nunes et al., 2017; Pinho et al., 2019). Yet, PQ-induced neurotoxicity has been reported to be distinct from other neurotoxins such as MPTP and rotenone (Richardson et al., 2005). To the best of our knowledge, our study is the first to demonstrate the protective effect of PS and TC against PQ-triggered PD-like motor and non-motor alterations in the zebrafish model.

Herein, we started our investigation with the determination of PQ LD_50_ after exposing the larvae to a series of PQ concentrations from 3 to 7 dpf. In the experimental conditions used, PQ resulted in the reduction of survival in a concentration- and time-dependent manner, and at 7 dpf, the LD_50_ value was determined to be approximately 500 μM. Thus, for further experiments with larvae, we used 500 μM PQ. This effect of PQ on survival is in agreement with a previous study, where PQ reduced the survival of zebrafish larvae in a concentration- and time-dependent fashion (Pinho et al., 2019). We next investigated the effect of 250 nM PS and TC on the survival of larvae after exposure to different concentrations of PQ (10–5000 μM) for different time periods (3–7 dpf). Interestingly, both L1 mimetics tended to improve the survival of zebrafish larvae at all concentrations of PQ (10–5000 μM), while the difference between percent survival of PQ *vs.* PQ plus L1 mimetics was not significant at any time point.

To characterize the *in vivo* Parkinsonian-like phenotype induced by PQ and its amelioration by L1 mimetics, we monitored their effects on spontaneous swim and sensorimotor reflexes in larvae. Swim activity is a sensitive parameter in monitoring the effect of toxins that can alter DA and serotonergic signaling (Sallinen et al., 2009; Irons et al., 2010, 2013). In our study, the PQ-treated group showed a reduction in the distance swum, both at 5 and 7 dpf, in comparison with the untreated control. Interestingly, the L1 mimetic-treated groups (PQ + PS/PQ + TC) that were exposed together with the PQ showed an improvement in distance swum in comparison with the PQ-treated group both at 5 and 7 dpf. The effect of PQ in our study on spontaneous swimming activity is in contradiction to a previous finding, where higher concentrations of PQ did not result in the reduction of distance swum (Pinho et al., 2019). The difference could be due to the differences in the evaluation of behavior (duration, light–dark conditions) used in the two studies. However, our findings are in agreement with Wang et al. (2018) who showed that 100 μM PQ resulted in a reduction in distance swum and swim velocity. Moreover, a 48-h (3–5 dpf) PQ treatment (250, 500, and 1000 μM) in the present study resulted in a concentration-dependent reduction in escape response evoked by either head- or tail-touch stimulation. Pinho et al. (2019) also reported a concentration-dependent reduction in escape response upon PQ treatment. Consistent with the protective effect of L1 mimetics in a spontaneous swim test, both PS and TC improved the escape responses either evoked by head- or tail-touch stimulation.

To further investigate the *in vivo* effect of PQ and the two L1 mimetics, we studied behavioral, biochemical, and functional parameters also in adult male zebrafish as PD is more frequent in males than in females (Cerri et al., 2019). Also, adult zebrafish are different when compared to larvae because of their well-developed central nervous system and complex behaviors (Stewart et al., 2015; Meshalkina et al., 2017). Here, we exposed adult male zebrafish to PQ *via* i.p. injections, which needed to be used as no locomotor alterations were observed when adult zebrafish were exposed to PQ (5 mg/l) in tank water (Bretaud et al., 2004). In this study, 20 mg/kg of PQ injection reduced distance swum, accompanied by an increase in the number of freezing events and freezing duration in the open field in comparison to the saline-injected control group. Moreover, the PQ-treated group showed anxiety-like behavior by swimming near the walls, while control saline-treated group explored the center of the tank. These findings are in agreement with previous studies (Bortolotto et al., 2014; Nunes et al., 2017; Müller et al., 2018). We observed that PS and TC attenuated the effect of PQ on behavior: In the PQ + PS and PQ + TC groups, the distance is improved in comparison to the PQ group. Also, co-treatment with L1 mimetics resulted in a decreased number of freezing bouts and freezing durations together with the prevention of anxiety-like behavior. Our findings are in line with our previous findings, where PS and TC ameliorated the locomotor deficits in both larvae and adult zebrafish models of spinal cord injury (Li et al., 2018; Sahu et al., 2018).

In addition to the motor symptoms resulting from the degeneration of DA neurons, several non-motor symptoms are also associated with PD (Chaudhuri and Odin, 2010). Therefore, we evaluated the effect of PQ and L1 mimetics on spatial memory using the T-maze task (Bortolotto et al., 2014). PQ treatment impaired the acquisition and consolidation of spatial memory, in agreement with the literature (Chen et al., 2010; Bortolotto et al., 2014). Of note, the response to novelty in the T-maze task could also be due to motor deficits and anxiety-like behavior induced by PQ. Zebrafish co-treated with L1 mimetic spent more time in the novel arm in comparison to the PQ-treated fish when their response to novelty was evaluated in the T-maze task, indicating an improvement of acquisition and consolidation of spatial memory. These findings are supported by the literature highlighting the improvement of learning and memory by PS and TC (Parent et al., 1999; Murakami et al., 2000; Yuede et al., 2007; Simpson et al., 2012). The improvement in the motor and non-motor deficits encouraged us to assess the neurochemical modifications upon co-treatment with PS or TC.

One of the critical enzymes in the synthesis of dopamine is TH, which catalyzes the hydroxylation of L-tyrosine at the phenol ring to yield 3,4-dihydroxyphenylalanine (DOPA) (Meiser et al., 2013). In our study, no significant change in TH expression in PQ-treated zebrafish or upon treatment with L1 mimetics was seen. Other studies with adult zebrafish did not also find a significant difference between the PQ-treated group in comparison with the control group (Bretaud et al., 2004; Bortolotto et al., 2014; Müller et al., 2018). However, many studies conducted in mice and rats show a reduction in the TH levels upon exposure to PQ (Brooks et al., 1999; Reeves et al., 2003; Ping et al., 2008; Buske and Gerlai, 2011; Breckenridge et al., 2013). Regarding differences in TH expression, they could possibly not have been detected with the method used. More sensitive methods for evaluating TH levels are quantitative immunohistochemistry, ELISA, or HPLC. Consistent with TH expression levels, we did not observe a significant reduction in the DA levels. Bortolotto et al. (2014) reported an increase in DA levels in adult zebrafish after long-term exposure to PQ, which is in contradiction to our findings. This difference may be due to methodological differences. As in another study, the authors reported a significant reduction (60%) in DA levels after exposure of 96 hpf embryos to 0.04 ppm PQ (Nellore and Nandita, 2015). The increase in the TH and DA levels upon co-treatment with L1 mimetics in our study is supported by previous findings where daily administration of PS (5 mg/kg i.p.) increased TH mRNA levels up to 71–115% in the rat locus coeruleus (Brady et al., 1992). In another study, a single intraperitoneal injection of PS elevated rat whole brain tyrosine levels (Matveychuk et al., 2014), which is a precursor for the production of the catecholamine neurotransmitters DA, noradrenaline, and adrenaline (Fernstrom and Fernstrom, 2007). MAOs are the mitochondrial enzymes that metabolize a range of amine substrates, including 5-HT and DA, and their abnormal activity has been implicated in various mental and neurodegenerative disorders (Wong et al., 2001; Bortolato and Shih, 2011). PS and TC are MAO inhibitors, and pharmacological inhibitors of these enzymes are of clinical benefit (Wong et al., 2001; Youdim et al., 2006).

Due to the high oxygen consumption rate and low anti-oxidant activity, the brain is more susceptible to oxidative stress in comparison to other tissues (Steinbrenner and Sies, 2009). Thus, excessive production of ROS or insufficient degradation leads to oxidative damage to astrocytes and neurons, resulting in acute brain injury and neurodegenerative diseases (Halliwell, 1992; Behl and Moosmann, 2002). Regarding the PQ, it is absorbed as PQ dication (PQ^2+^) by a potential-dependent carrier, which crosses the inner mitochondrial membrane (Cochemé and Murphy, 2008) where it serves as a potent redox cycler, resulting in the generation of superoxide, other ROS, which reduce the expression of antioxidant enzymes (Jones and Vale, 2000; Yumino et al., 2002; Thiruchelvam et al., 2005; Cochemé and Murphy, 2008), which further increase the sensitivity to PQ (Van Remmen et al., 2004; Yang and Tiffany-Castiglioni, 2005). However, PQ is a weak mitochondrial complex I inhibitor and thus acts most likely independent of complex I inhibition (Richardson et al., 2005). Another mechanism by which PQ generates ROS is through its interaction with glutamate, leading to excitotoxicity by depolarization and activation of NMDA and non-NMDA receptor channels. This overall process results in the induction of nitric oxide synthase, which further leads to mitochondrial dysfunction (Shimizu et al., 2003). The generation of ROS by PQ can further lead to alterations in bioenergetic parameters, such as the rate of oxygen consumption, electron transport chain activity, membrane potential, and impairment of ATP synthase activity (Drechsel and Patel, 2009; Blanco-Ayala et al., 2014). Therefore, considering the pro-oxidant effects of PQ, we characterized ROS levels, anti-oxidant enzyme activity (T-SOD), mitochondrial viability, and energy changes. PQ resulted in an increase in ROS levels, which is consistent with previous findings (Müller et al., 2018). In the present study, PS and TC prevented the increase in ROS levels. PS increased T-SOD levels in response to ROS generation by PQ while TC maintained T-SOD levels at the same level as in the control group. TC and PS protected the zebrafish brain from oxidative stress, which is in agreement with previous studies on the anti-oxidant properties of PS (MacKenzie et al., 2010; Bains and Hall, 2012; Ooi et al., 2015; Chen, Z. et al., 2016) and TC (Saxena et al., 2008; Karthivashan et al., 2017). Mitochondria are vital organelles, and their dysfunction is considered as an early event in neurodegenerative diseases, including PD (Chen and Chan, 2009; Subramaniam and Chesselet, 2013; Golpich et al., 2017). In line with the observed impaired mitochondrial viability in the PQ-treated group, ATP levels were found to be reduced upon PQ treatment, which is consistent with reduced mitochondrial viability upon PQ exposure (Nunes et al., 2017). Concerning ATP, a reduction in ATP levels has also been reported in PQ-treated 3 dpf larvae (Pinho et al., 2019). Reduction in the oxygen consumption rate after 24 h of PQ treatment in zebrafish larvae (Wang et al., 2018) could be attributed to the disruption and alteration of the electron transport chain and mitochondrial membrane potential, respectively (Castello et al., 2007; Cochemé and Murphy, 2008; Czerniczyniec et al., 2011). In the present study, PS and TC prevented the impairment of mitochondrial function and the reduction in the ATP levels. This beneficial function of PS and TC is supported by previous observations on the beneficial action of an L1 fragment, which is imported into mitochondria, where it interacts with the complex I subunit NDUFV2 (Kraus et al., 2018).

The L1 agonistic mimetic compounds PS and TC were discovered by screening small organic compound libraries for their structural and thus functional features by competitive ELISA (Kataria et al., 2016). The discovered compounds mimic the neurite outgrowth and neuronal survival-promoting functions of L1 by signal transduction as could be shown by experiments *in vitro*. *In vivo*, L1 mimetic compounds could be shown to enhance recovery from injury in a mouse model of spinal cord injury. Since PS and TC have reported functions other than being L1 mimetics, the ameliorating effects observed in the present study may well be due not only to the functions of the L1 mimetics but also to other functions reported for these compounds. These other functions could, when adequately dosed, add benefits to the encouraging effects of the L1 mimetic agonists in clinical settings.

## Data Availability Statement

The datasets generated for this study are available on request to the corresponding author.

## Ethics Statement

The animal study was reviewed and approved by the Animal Ethics Committee of Shantou University Medical College and by the Director of the Laboratory Animal Center.

## Author Contributions

TJ and MS designed the research. TJ, NJ, and LS performed the experiments. TJ and SS analyzed the data. TJ wrote the manuscript. SL edited the manuscript. MS finalized the manuscript.

## Conflict of Interest

The authors declare that the research was conducted in the absence of any commercial or financial relationships that could be construed as a potential conflict of interest.
